# Association Between the Use of Proton Pump Inhibitors and Severe Clinical Outcomes in COVID-19 Patients: A Retrospective Observational Study

**DOI:** 10.7759/cureus.72385

**Published:** 2024-10-25

**Authors:** Sharon Pinto, Hadia Al Lawati, Marwa Al Raisi, Balqees Al Maawali

**Affiliations:** 1 Primary Healthcare, Ministry of Health, Muscat, OMN; 2 Medicine, James Cook University Hospital, Middlesbrough, GBR; 3 Family Medicine, Ministry of Health, Muscat, OMN

**Keywords:** comorbid, covid-19, diabetes mellitus, hypertension, omani, proton pump inhibitors

## Abstract

Background

Proton pump inhibitors (PPIs) increase the risk of pneumonia secondary to PPI-induced hypochlorhydria. We aim to investigate the association between PPI and disease severity in coronavirus disease 2019 (COVID-19)-positive patients and the risk of hospitalizations in Muscat, Oman.

Methodology

COVID-19-positive patients aged 18 years and above at the time of diagnosis were included in this retrospective observational study. The details of the patients were retrieved from the electronic health records of the Al Shifa Hospital Information Management System and Tarassud. The composite primary endpoint was COVID-19 admission to a government tertiary hospital ward or intensive care within 14 days of diagnosis.

Results

A total of 506 COVID-19-positive patients were identified during the specified period. The mean age was 44 ± 15 years. The majority of the patients were Omani, and a female preponderance was observed. Overall, 104 (20.4%) patients were current PPI users. Admission due to COVID-19 was significantly associated with the presence of comorbid conditions such as diabetes mellitus (p = 0.001), hypertension (p = 0.001), and chronic kidney disease (p < 0.001). However, current PPI use (p = 0.140) was not significantly associated with an increased risk of hospitalization.

Conclusions

This data suggests that the use of PPIs during COVID-19 infection did not increase the risk of severe COVID-19 infection and poor outcomes leading to hospitalization in Muscat, Oman. However, the presence of other medical comorbidities, such as diabetes and hypertension, was associated with a higher risk of adverse clinical symptoms that resulted in hospitalization.

## Introduction

Coronavirus disease 2019 (COVID-19), a novel viral disease caused by severe acute respiratory syndrome coronavirus-2 (SARS-CoV-2) [1], emerged in December 2019 in Wuhan, China, and led to a global pandemic [2]. The typical incubation period for this virus ranges from 1 to 14 days, with peak infectivity occurring from two days before the onset of symptoms to seven days following symptom emergence. In the majority of instances, individuals exhibit mild-to-moderate symptoms, while 10-20% of cases present with severity that necessitates hospital admission. The admission rate to the intensive care unit (ICU) is observed to be 5%, attributed to conditions such as respiratory distress, sepsis, and multiorgan dysfunction. The associated mortality rate ranges from 2% to 4% [3]. In Oman, till May 22, 2020, a total of 6,370 cases were documented across the nation, with 1,821 individuals reported as recovered and 30 fatalities recorded, resulting in a mortality rate of 0.5% [4].

Angiotensin-converting enzyme 2 is a widely expressed receptor throughout the gut, kidneys, heart, testes, and brain and serves as a target receptor for SARS-CoV-2 [5]. Therefore, patients infected with the virus present with a myriad of symptoms, including respiratory and gastrointestinal (GI) symptoms such as diarrhea, nausea, and vomiting [6,7]. GI symptoms have been documented in 11.4% to 61.1% of individuals diagnosed with COVID-19 [8,9]. These symptoms can be mild or severe, requiring hospitalization and ICU admission. Several confirmed risk factors for severe COVID-19 illness included advanced age, diabetes mellitus, obesity, chronic pulmonary disease, chronic kidney disease, and cardiovascular disease [10].

In routine clinical practice, proton pump inhibitors (PPIs) are commonly prescribed drugs worldwide despite their potential adverse effects [11]. Dyspepsia and reflux are the most common indications for prescribing PPIs. PPIs decrease gastric acid secretion, which leads to increased pH in the stomach. This relatively high pH modulates the immune response by inhibiting the ability of neutrophils to eradicate infection [12,13]. In patients infected with COVID-19, hypochlorhydria resulting from the use of PPIs may result in continuous viral synthesis and replication in enterocytes, leading to a greater viral load [14].

A population-based study conducted in Sweden revealed that PPI use increased the overall risk of community-acquired pneumonia in 73% of the study participants [15]. In early 2020, a retrospective study of 295 hospitalized COVID-19 patients revealed a statistically significant association between prehospitalization PPI exposure and mortality due to COVID-19 [11]. A large cohort study conducted in South Korea concluded that subjects who consumed PPIs were at an increased risk of severe clinical outcomes due to COVID, such as admission to the ICU, intubation, and death (odds ratio (OR) = 1.90, 95% confidence interval (CI) = 1.46-2.77) [10].

Owing to the high morbidity and mortality rates of COVID-19, concerns regarding risk factors and the effects of different medications on disease susceptibility and severity have risen. In light of these concerns, this study aimed to evaluate the effect of PPIs on disease severity in COVID-19-positive patients in the Muscat governorate.

## Materials and methods

Data Source

Data were collected from two sources, namely, the Al Shifa Hospital Information Management System and Tarassud. Al Shifa is the electronic health record (EHR) system used by all Ministry of Health medical facilities in Oman from March 15, 2020, to November 15, 2020, covering primary, secondary, and tertiary care establishments. In response to the COVID-19 pandemic, Oman developed Tarassud, a comprehensive surveillance platform that provided real-time updates on COVID-19 statistics, polymerase chain reaction (PCR) test results, and information regarding tertiary hospital admissions. Access to the latter data was made available to physicians at their discretion.

The Ministry of Health in Oman provided gratis SARS-CoV-2 PCR testing to all citizens and residents exhibiting symptoms consistent with COVID-19 at government primary care health centers. Patients who underwent nasopharyngeal and oropharyngeal swab tests for SARS-CoV-2 PCR and subsequently tested positive between March 15, 2020, and November 15, 2020, were identified through the Laboratory module of the Al Shifa system across the following eight primary health centers: Muttrah, Hai Al Mina, Wadi Kabir, Ruwi, Wattayah, Al Khuwair (North), Ghubra, and Athaiba. The EHRs of all patients who tested positive during this study period were systematically reviewed. Collected patient information included age at diagnosis, sex, anthropometric measurements (body mass index, BMI), history of COVID-19 symptoms at presentation, past medical history, clinical examination findings, details of medications administered during the COVID-19 illness up to one month before the index date, and any referrals to tertiary care hospitals during the infectivity period. Additionally, data from Tarassud was utilized to document any visits to or admissions at tertiary hospitals during the infectivity period, along with the type of disposition. Any inpatient admission, whether to a COVID ward, high-dependency unit, or ICU, was classified as a hospital admission. Ethical approval for this retrospective observational study was obtained from the Directorate General of Health Services in Muscat (approval number: MOH/CSR/21/24311). Data collection was conducted in strict adherence to patient confidentiality and privacy regulations.

Study population

Adults older than 18 years of age who tested positive for SARS-CoV-2 by nasopharyngeal/oropharyngeal PCR swabs conducted at primary health centers from March 15, 2020, to November 15, 2020, were eligible for the study. Patients were excluded if they were pregnant during the infectivity period and/or if there was incomplete documentation in their respective EHRs (e.g., absent anthropometry, medical history, drug history, vital signs, clinical examination, or follow-up visits for de-isolation). Additionally, patients were excluded if they received most of their medical treatment for COVID-19 at primary health centers outside the Muscat governorate or non-Ministry of Health health facilities within Muscat due to a lack of access to their patient records.

Past medical illness was noted if it was documented at the first presentation and/or registered using an International Statistical Classification of Diseases and Related Health Problems 10th Revision code as a primary or secondary diagnosis at previous visits. Chronic medical illnesses included diabetes mellitus, hypertension, cardiovascular diseases such as ischemic heart disease and heart failure, stroke (both ischemic and hemorrhagic), chronic kidney disease, asthma, chronic obstructive pulmonary disease (COPD), and chronic lung disease. Current drug history, any past use of PPIs, chronic steroid use (defined as use for more than one month), and the use of other immunosuppressive drugs (including but not limited to methotrexate, monoclonal antibodies, current chemotherapy, and immunotherapy) were also noted.

Exposure

In Oman, the infectivity period of COVID-19 was 14 days from the index date. The index date was defined as the date of the first documented symptom before a positive SARS-CoV-2 PCR test for each patient. The study cohort was defined as all COVID-19-positive patients who used PPIs during the clinical course of their illness (1-14 days after their index date) or were on PPIs 1-31 days before their index date. Patients were classified as current PPI users if any PPI was consumed within 31 days before the index date or within 14 days of the index date. Additionally, a monthly repeated prescription of PPIs documented in their EHR at any government health facility was considered chronic use. However, any new prescription of PPIs after hospitalization during the infectious period was not considered current.

Non-users were patients who did not have a repeating prescription for PPIs, did not consume PPIs in their drug history, or consumed PPIs more than 31 days before the index date or after 14 days of the index date. A severe clinical outcome of COVID-19, in Oman, was defined as a patient with a positive COVID-19 PCR test who presented to the health center with severe symptoms of COVID-19 that necessitated hospital admission. The severe symptoms and signs included either oxygen saturation <92% on more than 6 L of oxygen, respiratory rate >30 per minute, use of accessory muscles, hypotension not responding to initial intravenous fluid boluses, and/or chest X-ray showing consolidation or new bilateral major infiltrates.

Outcomes

The primary endpoint of this study was admission to a tertiary government hospital of any duration within 14 days from the index date. The secondary endpoint for this study was the presence of clinical and radiological evident pneumonia.

Data analysis

Descriptive analysis was performed for the data. For continuous variables, the data were represented as mean ± standard deviation (SD), and for categorical variables, the data were represented as frequency and percentage. Pearson’s chi-square test was used to study the association between the variables. A p-value <0.05 was considered significant. SPSS version 23 (IBM Corp., Armonk, NY, USA) was used to analyze the gathered data.

## Results

In this retrospective observational study, a total of 506 COVID-19-positive patients who met the inclusion criteria were identified from March 15, 2020, to November 15, 2020. The majority of the patients were from Muttrah Health Center (n = 123, 24.3%), whereas the fewest were from Ghubrah Health Center (n = 24, 4.7%). The ages of the patients ranged from 18 to 87 years with female preponderance (n = 296, 58.5%). A small proportion of the patients were non-Omani (n = 22, 4.3%).

Approximately one-quarter of the patients were known to have diabetes (n = 129, 25.5%), and almost similar percentages were known to have hypertension (n = 161, 31.8%). A small proportion of the patients had cardiovascular disease (n = 27, 5.3%), stroke (n = 4, 0.8%), or chronic kidney disease (n = 27, 5.5%). Approximately half of the identified patients were obese, with a BMI greater than 30 kg/m^2^ (n = 232, 46.2%). Concerning diseases related to the respiratory system, 7.1% had asthma (n = 36), and even fewer had chronic lung disease (n = 4, 0.8%). Moreover, few patients were on steroids (n = 3, 0.6%). Among all identified patients, only one-tenth were admitted due to complications related to COVID-19 (n = 53, 10.5%). The demographics and clinical characteristics of the patients are demonstrated in Table 1.

**Table 1:** Demographics and clinical characteristics of COVID-19 patients (n = 506). The data are shown as frequency and percentage.

Variables	Values, n (%)
Age in years (mean ± SD)	45.21 ± 15.34
Gender (n %)	
Males	210 (41.5%)
Females	296 (58.5%)
Nationality	
Omani	484 (95.7%)
Non-Omani	22 (4.3%)
Body mass index (BMI) kg/m^2^ (mean ± SD)	26.12 ± 4.56
BMI >30 kg/m^2^, (n, %)	232 (45.84%)
Comorbidities (n, %)	
Diabetes	130 (25.7%)
Hypertension	161 (31.8)
Asthma	36 (7.1%)
Chronic kidney disease	28 (5.5%)
Cardiovascular disease	27 (5.3%)
Chronic lung disease	4 (0.8%)
Immunocompromised	5 (1%)
Chronic steroid use (n, %)	3 (0.6%)
Hospitalized (n, %)	53 (10.5%)

In this study, out of 506 COVID-19 patients, 104 (20.5%) were exposed to PPIs, while 402 patients were not exposed. There was no significant difference in age (p = 0.31), gender (p = 0.08), and BMI >30 kg/m^2^ (p = 0.54) between the PPI-exposed and unexposed patients. The incidence of comorbidities, diabetes mellitus (40.4% vs. 21.9%; p = 0.03), hypertension (44.2% vs. 28.8%; p = 0.02), and cardiovascular disease (11.5% vs. 3.7%; p = 0.007) was higher in COVID-19 patients exposed to PPI compared to unexposed patients. Meanwhile, there was no significant difference in hospitalization rate between the COVID-19 patients exposed and unexposed to PPI, but the proportion was higher in PPI users (14.4% vs. 9.4%; p = 0.65). A comparison of variables between the COVID-19 patients exposed to PPIs and those unexposed is presented in Table 2.

**Table 2:** Comparison of demographics and clinical variables in COVID-19 patients exposed and unexposed to proton pump inhibitor (PPI). The data are represented as mean ± SD, frequency (%). ^a^: unpaired Student’s t-test; ^b^: chi-square test; *: significant (p < 0.05); NS: non-significant

Variables	Total number (n = 506)	Exposed with PPI (n = 104)	Unexposed with PPI (n = 402)	P-value
Age in years (mean ± SD)	45.21 ± 15.34	44.65 ± 13.45	46.34 ± 15.12	0.31^aNS ^
Gender (N, %)				
Male	210	32 (30.8%)	178 (44.3%)	0.08^bNS^
Female	296	72 (69.2%)	224 (55.7%)	
BMI >30 kg/m^2^	232	48 (46.1%)	184 (45.8%)	0.54^bNS^
Comorbidities				
Diabetes mellitus	130	42 (40.4%)	88 (21.9%)	0.03^b*^
Hypertension	161	46 (44.2%)	116 (28.8%)	0.02^b*^
Cardiovascular disease	27	12 (11.5%)	15 (3.7%)	0.007^b*^
Stroke	4	1 (1%)	3 (7.4%)	0.08^bNS^
Chronic kidney disease	28	12 (11.5%)	16 (3.9%)	0.11^bNS^
Asthma	36	6(5.8%)	30 (7.5%)	0.43^bNS^
Chronic lung disease	4	2 (1.9%)	2 (3.9%)	0.87^bNS^
Immunocompromised	5	0	5 (0.9%)	0.43^bNS^
Chronic steroid use	3	2 (1.9%)	1 (0.2%)	0.52^bNS^
Hospitalization	53	15 (14.4%)	38 (9.4%)	0.65^bNS^

Hospitalization due to COVID-19 was associated with the presence of comorbid pathological conditions such as diabetes mellitus (p < 0.001), hypertension (p < 0.001), and chronic kidney disease (p < 0.001). In contrast, asthma (p = 0.67), BMI >30 kg/m^2^ (p = 0.14), cardiovascular disease (p = 0.08), COPD (p = 0.49), PPI use (p = 0.16), steroid use (p = 0.72), and immunocompromised status (p = 0.77) were not significantly linked to hospital admission. A comparison of the demographics and clinical factors associated with hospital admission in COVID-19 patients is presented in Table 3.

**Table 3:** Comparison of demographics and clinical factors associated with hospital admission in COVID-19 patients. The data are represented as frequency (%). Chi-square test, * denotes significance (p < 0.05). NS denotes non-significance.

Characteristics	No hospital admission (n)	Hospital admission (n)	P-value
Gender			0.182^NS^
Male	184	27	
Female	272	26	
Diabetes mellitus			<0.001^*^
No	352	27	
Yes	104	26	
Hypertension			<0.001^*^
No	322	25	
Yes	134	28	
Cardiovascular disease			0.082^NS^
No	435	47	
Yes	21	6	
Stroke			<0.001^*^
No	455	50	
Yes	1	3	
Chronic kidney disease			<0.001^*^
No	438	43	
Yes	18	10	
Asthma			0.67^NS^
No	425	48	
Yes	31	5	
Body mass index (BMI >30 kg/m^2^)			0.14^NS^
No	251	23	
Yes	205	30	
Chronic lung disease			<0.001^*^
No	455	50	
Yes	1	3	
Chronic obstructive pulmonary disease			0.49^NS^
No	455	52	
Yes	1	1	
Steroid use			0.72^NS^
No	454	52	
Yes	2	1	
Immunocompromised			0.77^NS^
No	449	53	
Yes	7	0	
Proton pump inhibitor use			0.16^NS^
No	376	38	
Yes	89	15	

## Discussion

The present study was conducted to investigate the relationship between the use of PPIs and the risk of adverse clinical outcomes in COVID-19-positive patients in Oman. Given the significant cultural differences across the world, we also aimed to identify possible associations between known risk factors and adverse clinical outcomes in COVID-19-positive patients.

In the present study, the rate of hospital admission did not significantly differ between the COVID-19 patients exposed and unexposed to PPI (14.4% vs 9.4%; p = 0.65). This finding is consistent with several other studies. A retrospective study conducted by Shah et al. involving 14,000 COVID-19-positive veterans in the United States reported no difference in primary (mortality and mechanical ventilation) and secondary composite endpoints (ICU and hospital admission) between PPI users and non-users [16]. Likewise, Yip et al. in Hong Kong reported no associations between adverse clinical outcomes and PPI consumption after their cohort was analyzed via propensity score weighting, propensity score matching, and multivariable adjustment for ICU admission (p = 0.54), mortality (p = 0.82), and use of invasive mechanical ventilation (p = 0.88) [17]. The present study also demonstrates that the proportion of comorbidities was higher in COVID-19 patients exposed to PPI compared to unexposed specifically for diabetes mellitus (40.4% vs. 21.9%; p = 0.03), hypertension (44.2% vs. 28.8%; p = 0.02), and cardiovascular disease (11.5% vs. 3.7%; p = 0.007). Similarly, a study conducted by Shupp et al. reported higher rates of diabetes (44.6% vs. 23.4%; p < 0.001), and cardiovascular disease (47.5% vs. 14.5%; p < 0.001) in active PPI users when compared to non-PPI users [18]. Likewise, Yao et al. concluded that COVID-19 cases with PPI exposure had a higher proportion of diabetes (18.20% vs. 13.3%; p = 0.001) and CVD (14% vs. 7.3%; p < 0.001) compared to non-PPI users [19].

We examined common comorbid conditions in this cohort. The most prevalent conditions were obesity (n = 235, 46%), hypertension (n = 162, 31.8%), and diabetes (n = 130, 25.5%). In the study, hypertension was significantly associated with admission due to COVID-19 (p = 0.001). In a study conducted in Oman, hypertension was associated with a two times increased risk of mortality in COVID-19 patients [20]. However, our study lacked data on chronic or inpatient medications for hypertension, preventing us from concluding its role in severe COVID-19. Additionally, this study demonstrated a significant association between diabetes (p = 0.001) and hospital admission due to COVID-19. A similar study conducted in Oman found that COVID-19 patients with diabetes were more likely to require critical care admission compared to those without diabetes (68.4% vs. 32.5%; OR = 1.5, p = 0.004) [21]. In the same study, patients with coronary artery disease had a 1.7-fold increase in critical care admission, which was statistically significant (15.83% vs. 3.6%; p = 0.04).

A study conducted in the United Kingdom found that COVID-19 patients with asthma experienced greater disease severity [22]. In contrast, this study found no significant association between asthma and severe COVID-19 (p = 0.43), which was similar to the findings of a systematic review and meta-analysis on the prognostic factors associated with mortality due to COVID-19 infection, which revealed that asthma was not associated with an increased risk for severe clinical outcomes or death [23]. A systematic review and meta-analysis reported that patients with end-stage chronic kidney disease on dialysis are at a higher risk of developing COVID-19 and experiencing increased mortality [24]. Additionally, we investigated the relationship between obesity and COVID-19 severity and found that a BMI greater than 30 kg/m^2^ was not associated with an increased risk of hospital admission (p = 0.54). This contrasts with findings from a prospective study conducted in the United Kingdom involving 6.9 million individuals, which demonstrated a linear increase in the risk of severe COVID-19, including higher hospital admissions and mortality rates when BMI exceeded 23 kg/m² [25]. Similarly, a study in Oman showed that COVID-19 patients with a BMI >40 kg/m² had a 2.8-fold increase in mortality [26].

Strengths and limitations

The strength of our study lies in it being the only study in the Middle Eastern region to analyze any association between COVID-19 severity and the use of PPIs. However, there were several limitations, including the possibility of measurement bias, as all our data were extracted from EHRs. Furthermore, as with any retrospective study, incomplete data are a major challenge. It was difficult to confirm the accuracy of some variables.

## Conclusions

Our data suggest that the use of PPIs during COVID-19 infection does not increase the risk of severe clinical outcomes leading to hospitalization, but further analysis is needed to determine the safety of PPIs among COVID-19-infected subjects. The presence of other clinical comorbidities, such as diabetes and hypertension, was associated with an increased risk of adverse clinical outcomes leading to hospitalization in Oman.
